# Dynamics of Resistance Development to Imatinib under Increasing Selection Pressure: A Combination of Mathematical Models and In Vitro Data

**DOI:** 10.1371/journal.pone.0028955

**Published:** 2011-12-22

**Authors:** Benjamin Werner, David Lutz, Tim H. Brümmendorf, Arne Traulsen, Stefan Balabanov

**Affiliations:** 1 Evolutionary Theory Group, Max-Planck-Institute for Evolutionary Biology, Plön, Germany; 2 Department of Oncology, Hematology, Bone Marrow Transplantation and Pneumology, Hubertus Wald Tumor Zentrum, University Hospital Eppendorf, Hamburg, Germany; 3 Department Hematology and Oncology, RWTH Medical School, Aachen, Germany; Roswell Park Cancer Institute, United States of America

## Abstract

In the last decade, cancer research has been a highly active and rapidly evolving scientific area. The ultimate goal of all efforts is a better understanding of the mechanisms that discriminate malignant from normal cell biology in order to allow the design of molecular targeted treatment strategies. In individual cases of malignant model diseases addicted to a specific, ideally single oncogene, e.g. Chronic myeloid leukemia (CML), specific tyrosine kinase inhibitors (TKI) have indeed been able to convert the disease from a ultimately life-threatening into a chronic disease with individual patients staying in remission even without treatment suggestive of operational cure. These developments have been raising hopes to transfer this concept to other cancer types. Unfortunately, cancer cells tend to develop both primary and secondary resistance to targeted drugs in a substantially higher frequency often leading to a failure of treatment clinically. Therefore, a detailed understanding of how cells can bypass targeted inhibition of signaling cascades crucial for malignant growths is necessary. Here, we have performed an in vitro experiment that investigates kinetics and mechanisms underlying resistance development in former drug sensitive cancer cells over time in vitro. We show that the dynamics observed in these experiments can be described by a simple mathematical model. By comparing these experimental data with the mathematical model, important parameters such as mutation rates, cellular fitness and the impact of individual drugs on these processes can be assessed. Excitingly, the experiment and the model suggest two fundamentally different ways of resistance evolution, i.e. acquisition of mutations and phenotype switching, each subject to different parameters. Most importantly, this complementary approach allows to assess the risk of resistance development in the different phases of treatment and thus helps to identify the critical periods where resistance development is most likely to occur.

## Introduction

The variety of approaches in cancer research ranges from clinical studies, experimental studies in vivo and vitro and genetic analysis to computational and mathematical models. Due to the insights from all these domains it was possible to develop specific targeted therapies for many cancer types and maybe cures for some are within reach [1]. A very promising example is the progress in treatment of patients with chronic myeloid leukemia (CML). CML is caused by a single reciprocal chromosomal translocation between the long arms of chromosome 9 and 22, the so called Philadelphia chromosome, in a hematopoietic stem cell [2]. The resulting BCR-ABL fusion gene encodes for a constitutive active tyrosine kinase, which is expressed and active in almost all haematopoietc cells and which leads to accelerated cell cycle activity [3]. As a result predominantly white blood cells at all stages of differentiation are significantly increased in peripheral blood and bone marrow of affected patients. Some years ago the standard treatment in the chronic phase was interferon-alpha and after disease progression chemotherapy with or without hematopoietic stem cell transplantation, a procedure that is associated with significant side effects and risks. The treatment algorithms changed due to the development of tyrosine kinase inhibitors (TKI) such as Imatinib [4], Nilotinib [5], Dasatinib [6] and Bosutinib [7]. These molecules bind specifically to the kinase domain of BCR-ABL and thereby strongly suppress the proliferation capability of CML cells. Since normal cells are less affected by this treatment, it allows normal hematopoiesis to be restored [8]. Long time clinical studies investigating the effect of TKIs show overwhelming success [4], [9], [10]. Unfortunately, there are cases in which patients do not respond to the drug or develop resistance during the treatment. This raises the question of how BCR-ABL positive cells bypass inhibition and develop resistance. A large number of in vivo and in vitro studies were performed, which identified different mutations causing resistance to TKIs [11], [12]. However, the spatial structure and thus the molecular mechanism of kinase domain binding various between different tyrosine kinase inhibitors and thus clones resistant to one inhibitor may be sensitive to an alternative inhibitor. Unfortunately, there are clones resistant to several inhibitors at the same time. For example the mutation T315I, the so called gatekeeper mutation, causes resistance to all approved TKIs. Agents as Ponatinib have been particularly designed to bind to such mutated kinase domains of BCR-ABL and suppress cell proliferation. These are in clinical trials and showing promising results [13]–[15]. Also many mathematical models concentrate on the probabilities and the dynamics of the development of such cross resistance and how to reduce the risk of resistance evolving to a minimum using combination therapies of different drugs [16]–[18]. We will not focus on this aspect in the following, but address the dynamics of resistance development instead.

Here, we discuss an experimental setup that induces resistance to Imatinib in initially sensitive BCR-ABL positive Ba/F3-p210 cells. In the following our aim is not to assess the probability of the occurrence of a specific resistance-conferring mutations nor to identify novel mutations, but to focus on the clonal dynamics that occurs during such an experimental setup. Instead, we show that not only mutations, but also intracellular processes that do not lead to any mutations can lead to the development of resistance. We investigate the average fitness of cancer cells exposed to a drug concentration that increases over time in this in vitro experiment. Basic features of this dynamic can be explained by a mathematical model, assuming a fitness of cancer cells that decreases with time as the drug concentration increases. By comparing the experimental data with simulation and analytical results we were able to assess individual parameters in this model and gain insights into the general cancer cell population dynamics under such environmental situations.

## Materials and Methods

### Materials

Imatinib was purchased from Toronto Research Chemicals Inc, Ontario, Canada. Stock solutions of IM (10 mg/mL; in DMSO/H2O (1∶1)) was stored at 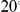
C. Murine BCR-ABL transduced pro-B cell line Ba/F3-p210 was obtained from N.P. Shah and C.L. Sawyers (UCLA, USA). Cells were cultured under standard conditions as previously described [19].

### Cell culture approach for induction of Imatinib resistance

In this experiment, Ba/F3-p210 cells carrying the BCR-ABL kinase were used as a model for resistance development to IM treatment, following a well established protocol [20]. Flow cytometry based cell sorting using a FACSAria (BD Biosciences) was employed to produce 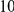 clones of BaF/3-p210 cells. These clones were expanded to cultures of 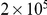 cells each. These cultures were exposed to Imatinib in a concentration increasing over time, starting with 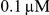. Cell viability was measured by the Trypan blue exclusion method [21]. The Imatinib concentration was periodically increased by 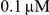 only if cell viability was above 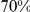, the concentration stays unchanged if the cell viability is between 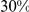 and 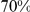 and withdrawn in case of cell viability less than 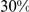. Thus the time the Imatinib concentration was increased differed for different clone lines, but the concentration was always increased within 12 days. Parallel, a control with untreated clones was performed. At the end of the experiment all Imatinib treated lineages were resistant to the drug, confirmed by a cytotoxicity MTT-assay.

### MTT assay

The 3-(4,5-dimethylthiazol-2-yl)-2,5-dipheny〈ltetrazoliumbromide (MTT) assay was performed as previously described [22]. In brief, BAF/3-p210 cells were plated into 96-well flat-bottomed microtiter plates (Becton Dickinson, Heidelberg, Germany) at 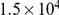 cells/well in 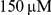 of their respective media. Cells were preincubated for 24 hours before increasing concentrations of Imatinib (0–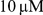) were added. All analyses were performed in triplicates. After 48 hours, the viable cells in each well were assayed. The dose-response effect for Imatinib at the point of inhibitory concentration (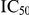) was analyzed by the median-effect method with CalcuSyn Software (Biosoft, Cambridge, United Kingdom) [23].

### Mutagenesis screen and real time PCR for BCR-ABL transcript

To identify possible BCR-ABL kinase domain mutations as mechanism of Imatinib resistance, the coding cDNA of the kinase domain was sequenced. For all RT-PCR reactions, total RNA was isolated using TRIzol (Invitrogen). cDNA was prepared by reverse transcription of 250 ng RNA with oligo(dT)15 and Superscript II reverse transcriptase (Invitrogen) and amplified using REDTaq ReadyMix PCR Reaction Mix (Sigma-Aldrich). BCR-ABL allele was amplified by nested PCR using BCR forward primer B2A(5′-TTCAGAAGCTTCTCCCTGACAT-3′) and ABL reverse primer 4065 (5′-CCTTCTCTAGCAGCTCATACACCTG-3′). Nested PCR reactions were performed using BCR F4 forward (5′-ACAGCATTCCGCTGACCATCAATA-3′) and reverse U396 (5′-GCCATAGGTAGCAATTTCCC). PCR products containing the kinase domain were sequenced with both forward primer 3306F (5′-TGGTTCATCATCATTCAACGG-3′) and reverse primer 4000R (5′-GGACATGCCATAGGTAGCA-3′). Real time PCR for BCR-ABL was performed as previously described [24], [25]. Amplification of ABL was used as a internal housekeeping gene.

### Quantification of MDR-1 on resistant clones

For the relative quantification of MDR-1 we used a flow cytometry-based assay. Cells were fixed in 2% formaldehyde for 10 minutes at 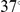
C, chilled on ice for 1 minute, and then permeabilized with ice-cold 90% methanol for 30 minutes on ice. From each sample, 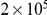 cells were washed with 2 mL incubation buffer (PBS/0.5% bovine serum albumin), centrifuged at 50 g for 5 minutes, and resuspended in 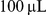 of incubation buffer with 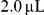 of a MDR-1 specific antibody (abcam, Cambridge, UK). After 45 minutes of incubation at RT, cells were washed twice, resuspended in 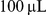 incubation buffer with 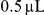 of the secondary antibody (anti-rabbit IgG FITC-conjugate; Jackson ImmunoResearch Europe, Newmarket, United Kingdom), and incubated at RT for 30 minutes in the dark. After washing two times with washing buffer cells were analysed using flow cytometry.

### Individual based model

**Figure 1:**
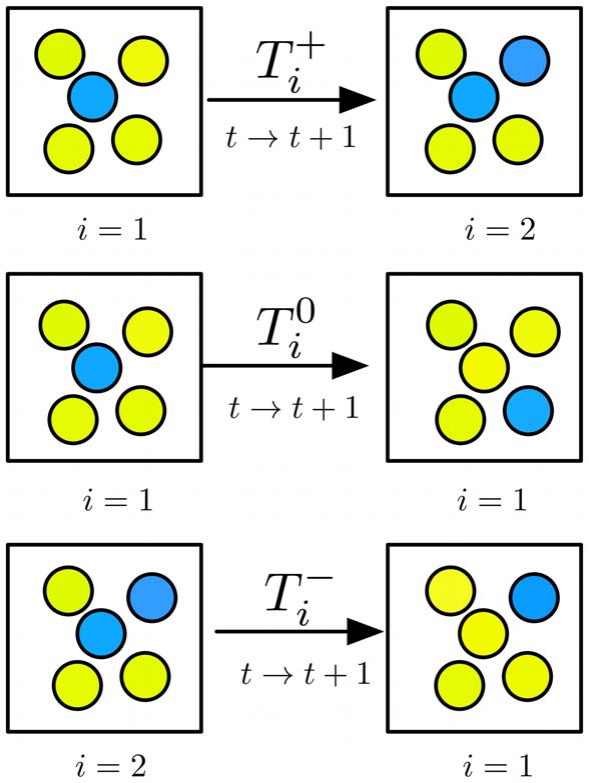
Individual based stochastic simulation as two type Moran process. We assume two possible cell types, wild type leukemic cancer cells sensitive to Imatinib (yellow) and resistant cancer cells (blue). In total, 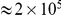 cells were included in the experiment and simulations. At time 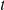 there are 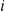 resistant cells and 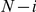 wild type cells. During the next time step 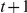 three cases are possible: (i) the number of resistant cells increases by one with probability 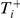, (ii) stays the same with probability 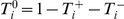 (iii) or decreases by one with probability 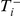, (see equation (2a) and (2b)).

We compare the dynamics in the mathematical model to the experiment based on the fitness of cells. We assume that the average fitness of the experimental cell population is the ratio between the number of vital cells and the total number of cells. Mathematically we model the cell population dynamics observed in the experiment by an individual based stochastic approach, applying a standard two type Moran process [26], [27]. We assume a population of 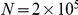 cells consisting of two subpopulations, wild type leukemic cancer cells sensitive to Imatinib and Imatinib resistant cancer cells. Initially, only wild type cancer cells are present, but transformations from wild types into the resistant type are possible. In each time step, one cell is chosen to reproduce and one cell is chosen to die, thus the total number of cells stays constant and the subpopulation sizes can change at most by 1 cell during one time step, see Fig. 1. If a wild type cancer cell is chosen to reproduce, an Imatinib resistant cancer cell is produced with a transformation probability 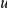. To avoid complications, we assume that resistant types cannot switch back to nonresistant types. This would not change the basic results, however. The two types differ in their fitness 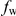 (for the wild type) and 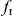 (for the resistant type) We assume a time dependent wild type fitness function, due to the increasing concentration of Imatinib in the experiment. We set the fitness of untreated wild types 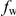 to one and assume that it decreases linearly with increasing Imatinib concentration,
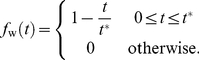
(1)


The parameter 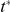 determines the impact of Imatinib on wild type cells. Choosing other decreasing functions, e.g. an exponentially decreasing function, does not change the results qualitatively. For the Imatinib resistant subpopulation we assume constant fitness, 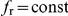. For 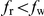, resistant cells proliferate slower than wild type cells, for 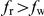 they proliferate faster than wild type cells. The experimental data suggest 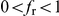 and thus initially resistant types would initially be out-competed by wild type cells, see below. Due to the decreasing fitness of wild type cells, we can go from one domain to the other in time. We assume that cell reproduction occurs proportional to fitness and simultaneously a random cell is chosen to die. Thus, the probability of increasing or decreasing the number of resistant cancer cells 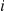 at time 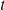 is notated by 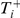 or 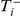 respectively,
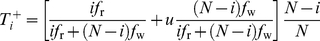
(2a)

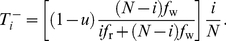
(2b)


The number of resistant cells stays unchanged with probability 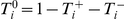. One generation consists of 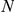 reproduction and death events, such that each cell reproduced on average once.

### Analytical approximation for large population size

If the population size 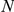 is large, we can describe the averages of our individual based model by a deterministic approach. In this case, we consider the frequency (relative population size) of the two cell types, wild type leukemic cells with frequency 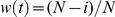 and Imatinib resistant cell types with frequency 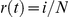. We use the same fitness functions as above and also assume transformations from the wild type to the resistant type. The average fitness of the system, which has been measured in the experiment, is given by 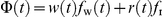. The change in wild type and resistant type frequencies for large populations are given by a replicator mutator equation [27], [28]

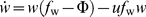
(3a)

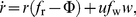
(3b)where the dot represents a derivative with respect to time 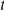. With the functions from above, these differential equations take the form
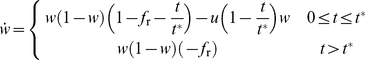
(4a)

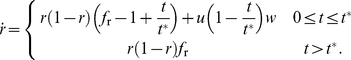
(4b)


These are time dependent nonlinear differential equations of the Bernoulli type, for which a general solution scheme exist [29]. Thus the system of differential equations can be reduced to integrals, which in this situation are solvable. Due to 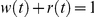, we only have to solve one of the equations. The general solution for 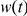 with respect to the initial condition 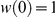 for 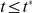 reads
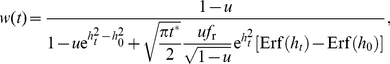
(5)where 
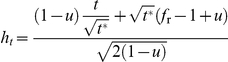
 and 
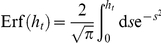
 is the error function. For 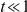, this can be approximated by
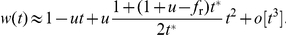



For 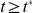, the number of resistant cells grows logistically and consequently, the number of wild type cells decreases logistically
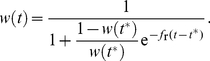
(6)


The average fitness of the total population becomes
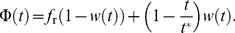
(7)


For small 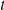, when 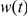 is approximately constant, the average fitness decreases approximately linearly determined by 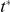.

## Results

### Development of Imatinib resistance in cell culture

**Figure 2:**
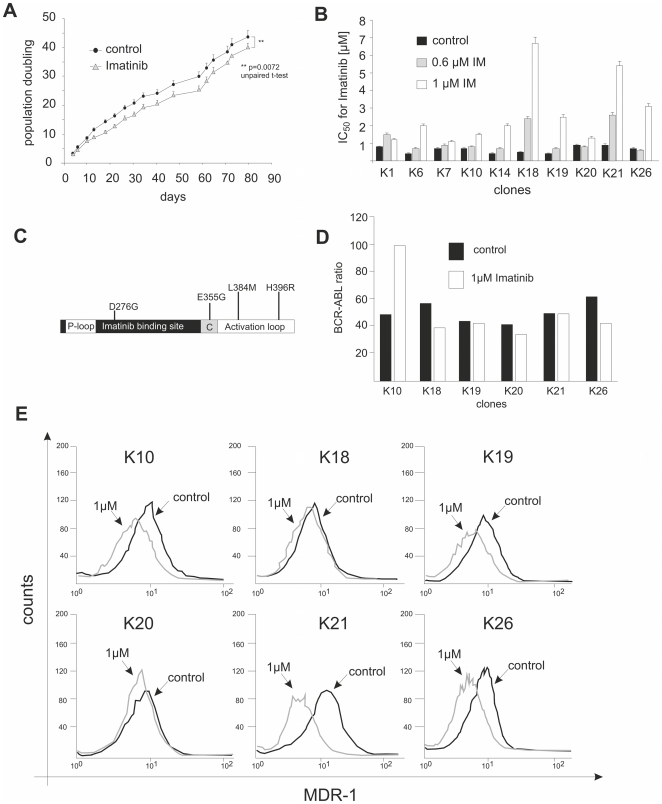
Experimental results. **A**) Average population doubling time observed for BAF/3-210 cells with (white triangles) and without (black dots) long-term Imatinib treatment. The number of population doubling for control cells was significantly higher compared to IM-treated cells. **B**) Measurment of 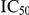 for Imatinib in control and resistant clones. The long-term presence of Imatinib increase the 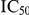 in all resistant cell clones. In particular, cell clones which were growing at 1 M Imatinib showed a marked increase of the 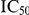 value. The cell viability was determined using MTT assay and 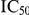 was calculated CalcuSyn software. **C**) Schematic representation of in vitro detected BCR-ABL mutations. **D**) Quantification of BCR-ABL transcript by reat-time PCR in resistant clones without BCR-ABL mutations. Only one clone (K10) exhibited an increased expression of BCR-ABL. **E**) Quantification of MDR-1 expresion by flow cytometry in resistant clones without BCR-ABL mutations. Imatinib resistance was not associated with an increased expression of MDR-1.

Using an in vitro approach to induce Imatinib resistance under increasing selection pressure, we detected resistance in 10 out of 10 BAF/3-p210 clones. As depicted in Fig. 2A the development of Imatinib resistance was associated with a significant decrease in the population doupling compared to control cells. The observed development of Imatinib resistance resulted in an significant increase of the 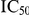 for Imatinib in all, but one clones (Fig. 2 B). For four resistant clones, it was possible to identify previously known resistance mutations in the BCR-ABL kinase domain. These were the D276G (prevents Imatinib from binding to BCR/ABL), E355G (blocks the TK catalytic centre) and L284M (inactivates the kinase) mutations (Fig. 2 C). No mutations of the kinase domain were found in the other clones, nevertheless they also developed resistance to Imatinib. In order to explore if the resistance in the non-mutated clones is based on an increased BCR-ABL expression we employed real time PCR for quantification of the BCR-ABL transcript. As depicted in Fig. 2 D we could only identify an up-regulation of BCR-ABL expression in one single clone. Furthermore, there was no increased expression of the ABC-transporter MDR-1 on resistance cell clones (Fig. 2 E). Based on these findings, we concluded that mechanisms other than kinase domain mutations, BCR-ABL over expression or MDR-1 up-regulation have lead to resistance in these cases.

### Fitting the mathematical model to the experimental data

**Figure 3:**
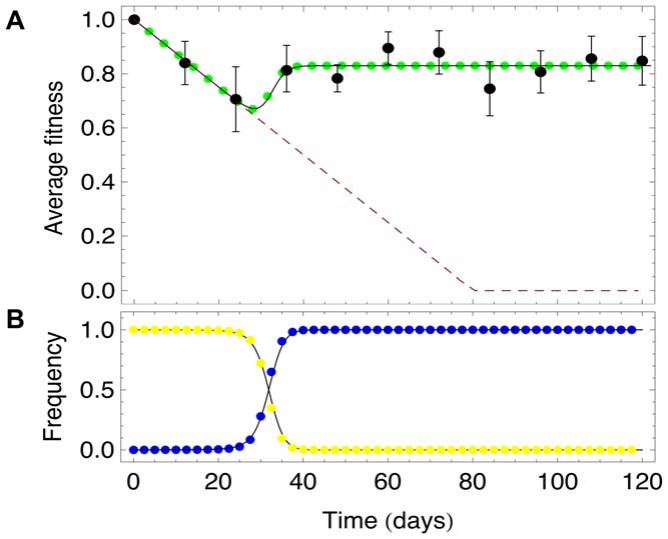
Dynamics of resistance development in the experiment and the mathematical model. **A**) Average fitness of the total population due to experiments (black dots), stochastic simulations (green dots) and analytical results (black line, due to equation (7)). The parameters of simulation and calculation were chosen to 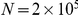, 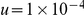, 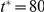 days and 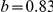. The dashed red line shows the linear decrease of the wild type fitness, the slope is determined by the critical time 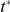. **B**) The average frequency of wild type cells due to simulation (yellow dots) and calculation (black line), as well as the frequency of resistant cancer cells (blue dots) over time. We always start with wild types only, but since the system selects for resistant cells, they fixate in the long run. This is why the average fitness of the total population exhibits a minimum. At the start of the experiments, the fitness of wild types decreases, but after some time resistant cells take over and the fitness increases until it saturates.

We now focus on the dynamics of the cell population. Initially, the average fitness of the total population decreases as the Imatinib concentration increased. At day 24 of the experiment, a global fitness minimum of 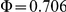 (experimental data) is obtained (Fig. 3 A). Subsequently, the average population fitness increased, although the environmental conditions become more challenging due to the ongoing increase of the Imatinib concentration. From day 48 until the end of the experiment the average fitness of the population was saturated and fluctuated around 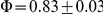. Our mathematical model offers a simple explanation for this result: In the beginning, only wild type cancer cells with decreasing fitness are present (Fig. 3 B). During the experiment, the frequency of resistant cell types increased and once they represent the bulk of the population, the average population fitness becomes independent of a further increase of Imatinib concentration. Consequently, the average fitness becomes constant. While it is challenging to analyze the underlying mechanisms of resistance evolution, we were able to adress the parameter ranges in the experiment based on our model.

First, we fixed time scales to make experimental and simulation results comparable. In the control experiment, untreated wild type cancer cells were grown in a regular growth medium and the population doubling times were recorded. We compare these doubling times to one generation in our simulation, which we define here as 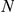 reproduction events in a population of size 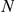. We found on average a doubling rate of 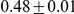 per day in the experiment, thus one generation in silico approximately relates to two days in vitro.

The initial fitness decrease was determined by the fitness decrease of wild type cells only and thus allowed us to fix the parameter 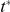 by linear regression of the average population fitness on day 0, 12 and 24, leading to 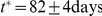. Thus, assuming a linear decrease of fitness according to equation (1) the wild type fitness would reach zero on day 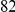 of the experiment (dashed black line in Fig. 3 A). Such an assumption of zero fitness is probably biologically not meaningful. However, our simulation results remain unchanged if we assume that the lowest possible fitness value of wild type cells is 0.5. This would be reached at day 40 of the experiment, where the resistant cell type already took over the population as shown in Fig. 3 B.

In our model the average fitness of the total population becomes constant at high Imatinib concentrations, because the resistant cell types take over the cell cultures. This fixed the average fitness of the resistant cell types to 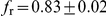 (averaged from day 48 on).

The only remaining free parameter is the switching probability 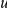, which is more difficult to assess. To determine 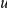, we fixed 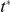 and 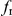 as discussed above and performed individual based stochastic simulations, which suggests that a switching probability between 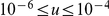 is compatible with the experiment. Smaller values of 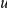 would cause a later increase in frequency of resistant cell types, but as soon as a certain fraction of resistant cells are present they fixate faster. For higher values of 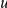 the minimum of the average fitness 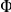 would be reached earlier. We also performed a non linear fit of equation (7) with the full solution for 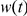, equation (5). The best fit yields a value of 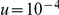. Note that 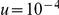 is too high to call this parameter a mutation rate. However, in the experiment only in 4 out of 10 samples mutations known for causing resistance to Imatinib were found.

**Figure 4:**
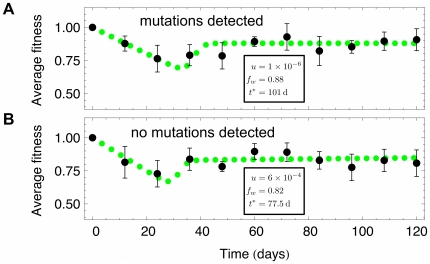
Average fitness observed for samples A) with and B) without detected mutations. The black dots are due to data and the green dots due to simulations. The evaluated parameters differ (see the inset of the plots), the fitness of the mutated cells is on average higher but their transformation rate is much smaller.

**Figure 5:**
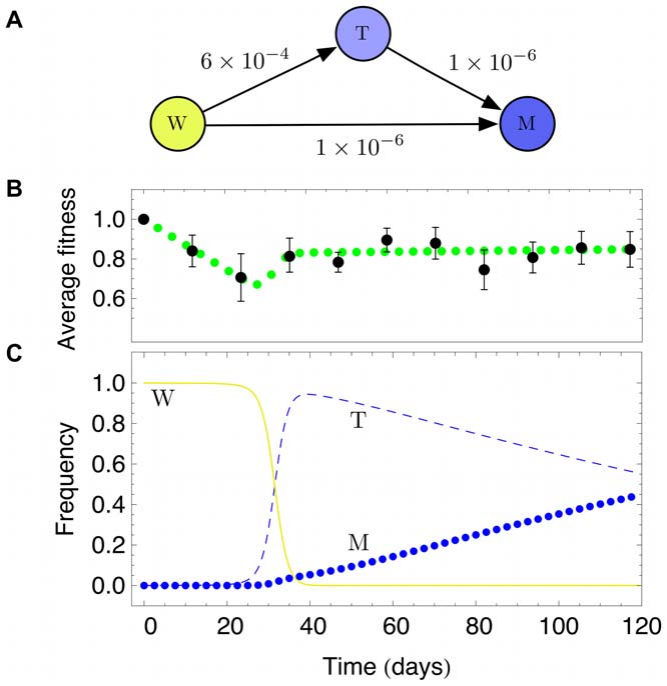
Stochastic simulations with three subpopulations. **A**) Transitions between the three types considered, wild types (W), resistant types without observed mutations (T) and resistant types with observed mutations (M). The switching probabilities are according to the arrows. **B**) Average fitness of the system described in a) due to simulations (green dots) and experimental data (black dots, as described in figure 3). The parameters are those observed in figure 4. **C**) Average frequency of the three types. Initially, only wild types (yellow line) are present, at day 37 of the experiment resistant types without observed mutations (T, dashed blue line) reach almost fixation, but in the long run mutated types (M, blue dots) take over due to their fitness advantage. At day 120 of the experiment we have 0.57 T types and 0.43 M types (6 T and 4 M types were observed in the experiment at day 120).

To take these two different experimental outcomes into account, we performed the procedure described above independently for samples with and without mutations. The result is shown in Fig. 4. In samples where mutations were detected, we observe 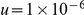, 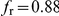. In the case of no detected mutations, we found 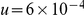 and 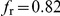. Thus, the transformation probability differs by magnitudes and samples with mutations seemed finally fitter than samples without mutations. This leads to the possibility that two mechanism may cause resistance, mutation and a sort of phenotype switching. We extended our model to incooperate these two distinct effects, as shown in Fig. 5. With this extension, wild type cells switch into mutated cells (M) with probability 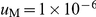 and fitness 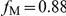. Alternatively, they switch into transformed types (T) with probability 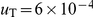 and fitness 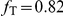. Additionally, transformed types (T) can mutate with probability 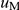 into mutated types (M). The dynamics for this scenario is shown in Fig. 5 C. Initially only wild type cells are present in the simulation. Due to the higher switching probability transformed types take over almost the whole population, but due to their fitness advantage and the directed mutations in the long run the mutant types fixate. However the experiment was stopped after 120 days. In the experiment we found mutant types in 4 out of 10 samples. At this time we observe frequencies of 0.43 for mutated types and 0.57 for transformed types in the simulation, compatible with the experiment. Also in the simulation, the average fitness of the population goes through a minimum, but the fitness still increases after 120 days, because the mutant type does not reach fixation yet.

## Discussion

Resistance to TKI is a frequent clinical problem in treatment of CML patients. The appearance of resistant cell clones undermines our abillity to control the malignant BCR-ABL positive cell clone and thereby prevent the successful treatment of CML patients. The mechanisms of TKI resistance are either BCR-ABL dependent or BCR-ABL independent [30]. Mutations in the BCR-ABL kinase domain represent the most frequent BCR-ABL dependent mechanisms. On the other hand, overexpression of BCR-ABL and overexpression of drug transporters are known kinase independent mechanisms of TKI resistance. The rise of drug resistance is a evolutionary process characterized by a competition between sensitive and resistant cells but not an active process of resistance induction by the drug [31].

Using a combination of a clonal in vitro approach for induction of Imatinib resistance and mathematical modeling, we were able to explain the dynamics of the average fitness of Imatinib treated cancer cell populations in vitro in a simple model assuming that:

- The population consists of only three subpopulation, the wild type cancer cells (the only cell type present at the beginning of the experiment) and resistant cell types (mutated and transformed cells).
- The fitness of wild type cells decreases with increasing drug concentration over time. The fitness of resistant types is lower than the wild type fitness, but both are constant.
- We introduced directed transformation. Wild types transform into resistant types, the reversal is neglected.

With these assumptions, we provided a method to fix the model parameters and thus get some insights into the mechanism that cause the dynamics. Comparing the analytical solution with experimental results enabled us to fix important parameter of the system. Accordingly, it is possible to derive a range for the transformation probability 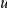, which is 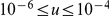 for this experimental setup. This is high compared to values normally assumed for mutations, which seems to be about 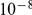 for normal cells. But the mutation rate of malignant cells has been reported to be at least 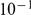 to 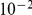 higher [32], [33]. If we perform our analysis separately for the samples with and without mutations, we find different parameter values. For the mutated cell lines, we found 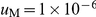, which is in the expected range for mutation rates of such malignant cells. But for samples without mutations, we find 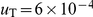. This supports the idea that not only mutations are responsible for drug resistance evolution in this case, but that also other mechanisms are likely to be involved [34]. Noteworthy, we could not detect BCR-ABL- or MDR-1 overexpression as the reason for resistance. However, there is some evidence that an activation of additional pathways, like the phosphatidylinositol-3-kinase (PI3K)-AKT pathway [20] or Src family kinase [35], [36], can compensate for the BCR-ABL inhibition and can promote the proliferation of these cells in the presence of Imatinib. From our mathematical model and our experimental results, one can conclude that particularly the time frame between day 24 and day 48 is crucial for development of resistance. It can be speculated that starting with day 24, the cells launch out to adapt their metabolism to the increasing pressure of raising Imatinib concentrations. This kind of adaptions are normaly accompanied by an activation of novel signaling pathways and can drive the cellular metabolism of resistant cells to an increased glycolytic activity and phospholipid turnover [37], [38]. In this context, our model represents a basis to determine the critical time point and Imatinib concentration for further studies to detect novel BCR-ABL independent resistance mechanisms. This should be done by a comprehensive and integrative genome-, transcriptome-, proteome- and metabolome analysis and may lead to additional insights into activated pathways of resistant CML cells. Recent in vitro and in silico studies have revealed that a combination of drugs with different mode of actions can prevent treatment failures due to drug resistance [16]–[18], [39]. However, the appropriate targets are not defined and must be elucidate in further studies.

From our study, it is apparent that the development of Imatinib resistance is associated with reduced fitness of resistant cells in vitro. Translating this into fitness in vivo can be challenging, due to additional aspects as the hierarchical structure of hematopoiesis [40]–[42], failed targeting of cancer stem cells, for example due to quiescent cells, [43], [44] and possible aging effects [45]. Furthermore very small differences in vitro can cause important measurable differences in vivo, but also in this case mathematical approaches can be helpful [46]. Notably, the fitness of clones harboring a BCR-ABL mutation was higher compared to the unmutated clones. This might be due to the proposed switch on alternative or additional programs in unmutated clones which may be costly in terms of proliferation, but enable the cell to function independent of the BCR-ABL kinase. Based on recent studies, it can be speculated that in mutated clones the mutations are preexisting and the cells do not need to adapt their metabolism to reach resistance [47]. In this case the reduced fitness reflect the differences between mutant and wild-type BCR-ABL. Recent studies have revealed that certain mutated BCR-ABL kinases are accompanied by reduced oncogenic activity [48], [49]. These findings are supported by clinical studies. Hanfstein et al. have shown a deselection of mutant BCR-ABL positive clones after cessation of tyrosine kinase inhibitor [50], as also suggested by mathematical models [8], [51]. Fitness costs are commonly associated with drug resistance. Particularly, resistance against antibiotics is frequently observed in vitro and in vivo and is associated with reduced fitness of resistant clones [52], [53]. In comparison to that, the existence of a fitness cost in resistance to cancer drugs has not been analysed in detail yet. Therefore, our data represent an appropriate model to further investigate in this cellular behavioral pattern.
